# Host–parasite genotypic interactions in the honey bee: the dynamics of diversity

**DOI:** 10.1002/ece3.599

**Published:** 2013-06-06

**Authors:** Sophie E F Evison, Geraldine Fazio, Paula Chappell, Kirsten Foley, Annette B Jensen, William O H Hughes

**Affiliations:** 1 School of Biology, Faculty of Biological Sciences, University of LeedsLS2 9JT, U.K; 2 Center for Social Evolution, Department of Agriculture and Ecology, University of CopenhagenThorvaldsensvej 40, DK 1871, Frederiksberg C, Denmark; 3 School of Life Sciences, University of SussexBrighton, BN1 9QG, U.K

**Keywords:** *Apis mellifera*, *Ascosphaera apis*, *Aspergillus flavus*, disease resistance, genetic diversity, Polyandry, social insect

## Abstract

Parasites are thought to be a major driving force shaping genetic variation in their host, and are suggested to be a significant reason for the maintenance of sexual reproduction. A leading hypothesis for the occurrence of multiple mating (polyandry) in social insects is that the genetic diversity generated within-colonies through this behavior promotes disease resistance. This benefit is likely to be particularly significant when colonies are exposed to multiple species and strains of parasites, but host–parasite genotypic interactions in social insects are little known. We investigated this using honey bees, which are naturally polyandrous and consequently produce genetically diverse colonies containing multiple genotypes (patrilines), and which are also known to host multiple strains of various parasite species. We found that host genotypes differed significantly in their resistance to different strains of the obligate fungal parasite that causes chalkbrood disease, while genotypic variation in resistance to the facultative fungal parasite that causes stonebrood disease was less pronounced. Our results show that genetic variation in disease resistance depends in part on the parasite genotype, as well as species, with the latter most likely relating to differences in parasite life history and host–parasite coevolution. Our results suggest that the selection pressure from genetically diverse parasites might be an important driving force in the evolution of polyandry, a mechanism that generates significant genetic diversity in social insects.

## Introduction

The importance of genetic diversity in biological systems has been a key topic in evolutionary biology for more than 80 years (Fisher 1930). Nowhere is this more apparent than in the interactions between hosts and their parasites. The coevolutionary arms race that arises between hosts and parasites relies on genetic variation in both host resistance and parasite virulence (Carius et al. 2001). Genetic diversity within a host population is predicted to reduce prevalence of parasites and disease intensity (Leonard 1969; Hamilton 1987; Sherman et al. 1988; Schmid-Hempel 1998). Here, the selective advantage of individuals containing rare genes for resistance to a parasite can promote sexual reproduction and the production of more diverse offspring with rare resistance genes (Hamilton, 1980). Many studies have shown that parasite virulence and fitness depends on host genotype as well as the genotype of the parasite, with some hosts being more susceptible or resistant to a particular parasite than others (Ebert and Hamilton 1996; Carius et al. 2001). Homogeneous host populations that are composed entirely of resistant individuals will have the lowest infection levels if there is no variation in parasite virulence (Boomsma and Ratnieks 1996). However, the advantage of host genetic diversity depends on variation in parasite genotype, an aspect of host–parasite interactions that is not addressed explicitly in most empirical studies (Ganz and Ebert 2010).

Colonies of social insects are characterized by their dense aggregations of related individuals in homeostatic nest environments, factors that may significantly increase the risk of disease outbreaks (Schmid-Hempel 1998). As a consequence, they use a combination of both physiological and behavioral mechanisms to combat disease. Genetic variation underlying the ability of honey bees to mount an individual immune response has been suggested to be lower than that of solitary insects (Evans et al. 2006), but behavioral defenses such as hygienic behavior may compensate for this deficit (Spivak and Gilliam 1998; Crozier and Fjerdingstad 2001; Wilson-Rich et al. 2009; Oxley et al. 2010). The vulnerability of low genetic diversity groups to parasites is very likely to represent an important selection pressure on social insect hosts to evolve mechanisms that increase intracolonial genetic diversity in order to promote disease resistance, both at the individual and the colony level.

Polyandry, the insemination of females with sperm from multiple males, is a mechanism that generates significant genetic diversity in nature. Polyandry is taxonomically widespread in the animal kingdom, but is hard to explain because of the apparent costs involved, such as increased exposure to sexually transmitted diseases, higher risk of predation, and harm from males (Jennions and Petrie 2000; Crozier and Fjerdingstad 2001). These costs may be particularly high in social insects because it occurs during the riskiest period of a queen's life (the mating flight, where the queen leaves the nest to mate and is not protected by workers; Weber 1972; Fowler et al. 1986; Baer et al. 2006). The genetic diversity generated through polyandry, however, has been suggested to improve the disease resistance of colonies and therefore outweigh the costs involved (Hamilton 1987; Sherman et al. 1988; Brown and Schmid-Hempel 2003). Although genetically diverse populations may be vulnerable to a larger selection of parasite strains (Van Baalen and Beekman 2006), higher genetic diversity can also make host populations less susceptible to parasites by increasing the chances of rare genotypes that provide resistance alleles (Schmid-Hempel 1998; Boomsma et al. 2005). Evidence for this comes from social insects (bumblebees: Baer and Schmid-Hempel 1999; honey bees: Tarpy 2003; Seeley and Tarpy 2007; leaf-cutting ants:, Hughes and Boomsma 2004, 2006; Hughes et al. 2010; wood ants: Reber et al. 2008; Armitage et al. 2011), and from other animals, from water fleas to humans (Lloyd-Smith et al. 2005; Altermatt and Ebert 2008) and also from plants (e.g., Chung et al. 2012).

Here, we investigate host–parasite interactions between the honey bee *Apis mellifera* and its fungal brood parasites *Ascosphaera apis* and *Aspergillus flavus* (the causative agents of chalkbrood and stonebrood disease, respectively). The honey bee, *A. mellifera*, is particularly suitable for examining host genetic variation because reproductive females are highly polyandrous; colonies contain a single mother queen mated with 12 ± 8 (haploid) males (Tarpy et al. 2004). Their female (diploid) worker offspring thus consist of a number of full-sister lineages (patrilines) that differ only in their paternal genotype because they share the same rearing conditions, maternal cues and maternal genotype on average. Hence, they provide an ideal system to assess not only the underlying mechanisms behind the potential benefits of polyandry in terms of disease resistance but also genotypic interactions with the parasites that cause disease. We were particularly interested in the variation in the response of different host genotypes to their obligate parasite *Asc. apis*, which will have coevolved with honeybees, and therefore we examined the response to three different strains. For comparison, we also examined the host responses to a single strain of the ubiquitous fungus *Asp. flavus* that is an opportunistic parasite with a looser evolutionary history with the honey bee (Foley et al. 2012), which we predicted would be associated with weaker host genetic variation in resistance.

## Materials and Methods

### Collection and *in vitro* rearing of larvae

We collected larvae from four colonies of the European honey bee *A. mellifera,* each headed by unrelated, naturally mated queens (Colonies 4, 5, 8, and 44). Larvae were reared individually in 48-well tissue culture plates on a diet of 50% royal jelly, 6% d-glucose, 6% d-fructose and sterile deionized water, following a modified version of the procedures described by Aupinel et al. (2005) and Jensen et al. (2009). One to 2-day-old larvae were removed from the comb using a Swiss grafting tool (Swinty, Sønderborg, Denmark) and transferred onto a droplet of larval diet within a cell culture plate. The plates were then placed in sealed boxes containing a pool of 0.04% K_2_SO_4_ in order to establish high relative humidity and maintained at 34°C. Larvae were fed daily *ad libitum* until they began to defecate (after molting to the 5th instar); the wells were then cleaned with a cotton bud.

### Treatment of larvae and observation of mortality

Spores were harvested from media plates of three different strains of the heterothallic fungus *Asc. apis*, each formed by the mating of two isolates (strain I by isolates ARSEF 7405 + 7406; strain E by isolates KVL 0798 + 06117 and strain F by isolates KVL 06123 + 06132) and one strain of *Asp. flavus*, all obtained from culture collections kept at the University of Copenhagen. Spore suspensions were made by grinding ∼0.01 g of spore material in a glass tissue homogenizer with 50-μL deinonized water. Released spores were made up to a volume of 1 mL with sterile deionized water and left to stand for 20 min so that the asci settled out. A 0.5-mL aliquot of the resulting medium-density spore solution was taken and stored in a separate eppendorf tube. The concentrations of the spore solutions were determined using FastRead disposable hemocytometer (Immune Systems, UK) and solutions were diluted to the following concentrations; *Asp. flavus*: 1.0 × 10^5^, *Asc. apis* strain I: 5.0 × 10^5^, *Asc. apis* strain E: 3.75 × 10^6^, and *Asc. apis* strain F: 1.95 × 10^6^ spores per mL to account for differences in spore viability (which was determined as detailed in Vojvodic et al. 2011). Spore suspensions were applied directly to the mouth of larvae in 5 μL doses 2 days after grafting (or 5 μL sterilized water in the case of control larvae), and mortality and evidence of infection (hyphal growth) was monitored daily for 9 days using a stereo microscope.

### Genotyping

Larvae that died from *Asc. apis* or *Asp. flavus* infections as well as the larvae that survived the 9-day period after infection were genotyped. Larvae that died due to other causes showed rapid bacterial decomposition, which made them unsuitable for DNA extraction and were therefore excluded. This was the case for all of the control larvae that died and for a similar proportion of larvae in each of the treatments (Colony 4: treatment = 28%, control = 19%; Colony 5: treatment = 39%, control = 26%; Colony 8: treatment = 21%, control = 24%; Colony 44: treatment = 18%, control = 19%), so the exclusion of these decomposed larvae did not confound the results. All of the remaining control larvae (1208 of 1546) survived to the end of the experiment and so were not genotyped because they by definition did not have any patriline or colony variation in survival. Larvae were genotyped at eight microsatellite loci: A7, A29, B124, A35, A79, A107, A014 (Estoup et al. 1994), and AP243 (Solignac et al. 2003). Total DNA was extracted from 5 to 30 mg of dried larval tissue using 30–100 μL of a 5% Chelex® (Bio-Rad, Berkeley, CA) 100 Resin (200–400 mesh – Sodium form) solution in water. After 15 min at 99°C and 20 min centrifugation at 4600 rpm, 1 μL of the supernatant was used in each of two multiplex PCRs containing 0.2 μmol/L each primer (Multiplex A: A7, A29, B124, AP243; Multiplex B: A79, A107, A14, A35), 250 μmol/L dNTPs, 0.8 units of GoTaq Polymerase (Promega Corporation, Madison, WI) in a final volume of 15 μL. The thermocycling profile for Multiplex A was 94°C for 3 min, five cycles at 94°C for 30 sec, 60°C to 55°C (1°C drop per cycle) for 45 sec, and 72°C for 45 sec, further 30 cycles with annealing at 55°C and a final extension at 72°C for 7 min. The thermocycling profile for Multiplex B was 94°C for 3 min, two cycles at 94°C for 30 sec, 62°C for 45 sec, and 72°C for 45 sec, two cycles using 60°C as annealing temperature, two cycles using 58°C as annealing temperature, and 30 cycles using 54°C as annealing temperature with a final extension at 72°C for 7 min. All forward primers were fluorescently labeled to allow detection in a 3130*xl* Genetic Analyzer (Applied Biosystems, Madison, WI). Allele sizes were scored by comparison with internal size markers using Genemapper® v3.7 software (Applied Biosystems, Foster City, CA). Multilocus offspring genotypes were used to deduce the genotypes of colony queens and their multiple mates, and the workers were assigned to patrilines within their colony with extremely low detection errors (0.0001%; Boomsma and Ratnieks 1996).

### Statistical analysis

All analysis was carried out using R statistical software (R Development Core Team 2012). Differences in survival of larvae between treatments and patrilines within-colonies and their interaction were analyzed using Cox-proportional hazard survival models implemented using the coxph function of the survival package (Therneau 2011), with survivors of the experiment incorporated as right-censored data. As a measure of effect size to allow us to control for both variable sample sizes within treatments and patriline numbers between colonies we calculated the hazard ratio for each patriline as compared to its own colony's control survival. The effect of colony on hazard ratio was then assessed using a mixed-effects model, implemented using the lmer function in the lme4 package (Bates and Maechler 2010), with patriline fitted as the random term.

## Results

### Host and parasite genotypic interactions

**Figure 1:**
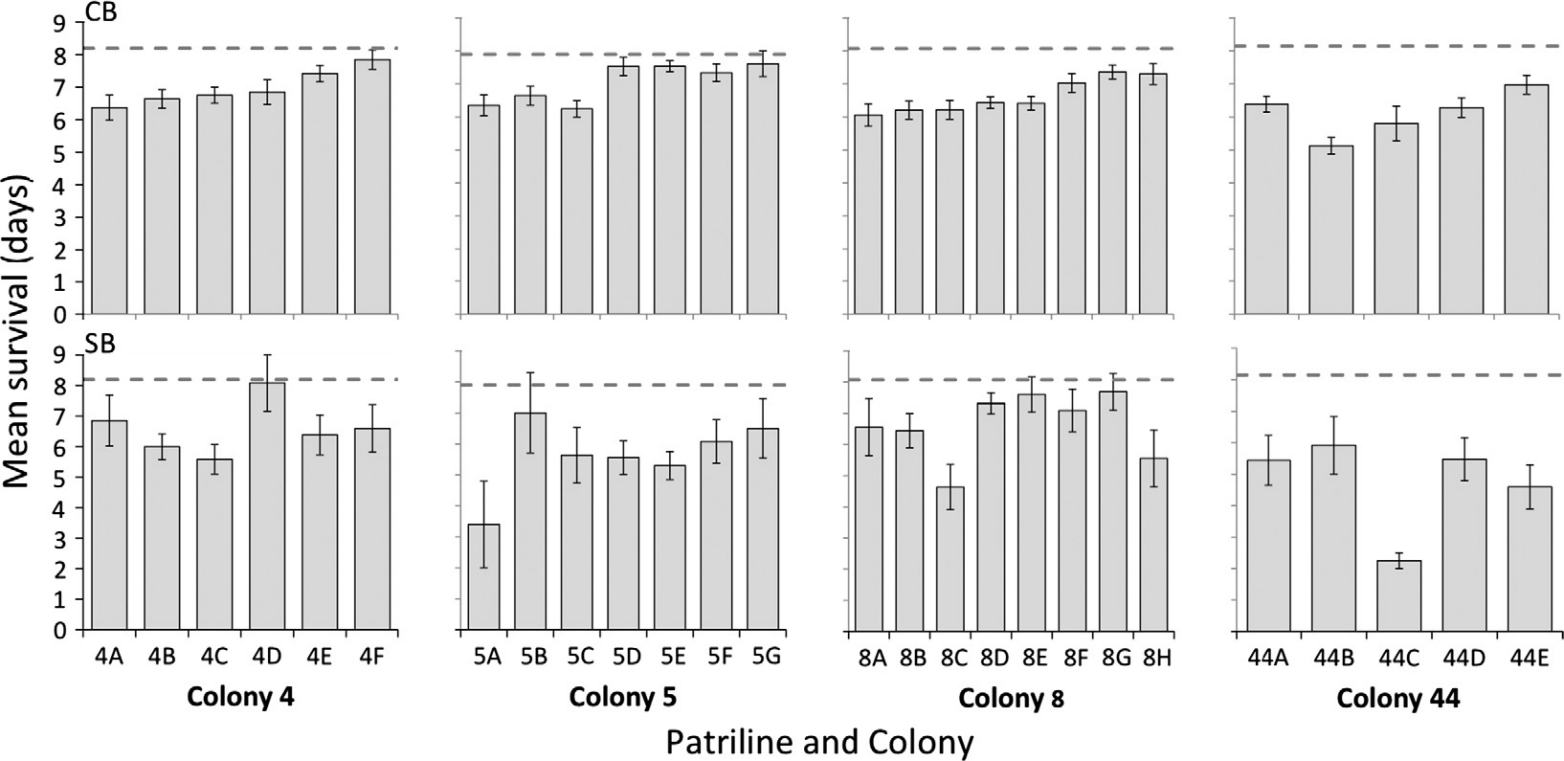
Mean ± SE survival time in days of each patriline within the four colonies (columns) split by parasite (rows); SB, stonebrood, *Asp. flavus* (bottom row); CB, chalkbrood, *Asc. apis* (top row). Only patrilines containing more than three individuals per patriline per treatment are shown. The dashed line represents mean control survival level of that colony.

We first wished to establish if there were genotypic differences in survival after infection by our two parasite species. We found a significant interaction between the species of parasite infecting the host and host patriline in three of four colonies, indicating that host genotypes of these colonies varied in their relative susceptibility to the different parasite species (Fig. 1; Table 1 row b). When we analyzed the effects of each parasite species on each colony separately we found genotypic differences in survival of larvae exposed to the chalkbrood parasite in all four colonies, but only in the larvae of one colony when exposed to the stonebrood parasite (Table 1 row c, d).

**Table 1:** Statistical results of the different survival analyses performed on survival data from each colony

	Colony 4	Colony 5	Colony 8	Colony 44
(a) Parasite species effect	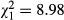 *P* = 0.003	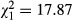 *P* < 0.001	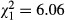 *P* = 0.014	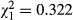 *P* = 0.571
(b) Parasite species × patriline interaction	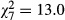 *P* = 0.071	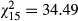 *P* = 0.003	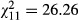 *P* = 0.006	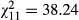 *P* < 0.001
(c) Stonebrood patriline effect	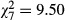 *P* = 0.218	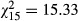 *P* = 0.428	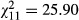 *P* = 0.007	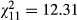 *P* = 0.341
(d) Chalkbrood patriline effect	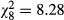 *P* = 0.032	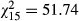 *P* < 0.001	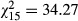 *P* = 0.003	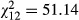 *P* < 0.001
(e) Chalkbrood strain effect	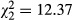 *P* = 0.002	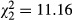 *P* = 0.004	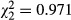 *P* = 0.615	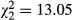 *P* = 0.001
(f) Chalkbrood strain × patriline interaction	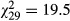 *P* = 0.191	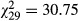 *P* = 0.377	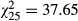 *P* = 0.050	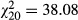 *P* = 0.009
(g) Chalkbrood strain E patriline effect	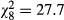 *P* < 0.001	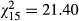 *P* = 0.125	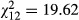 *P* = 0.075	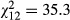 *P* < 0.001
(h) Chalkbrood strain F patriline effect	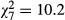 *P* = 0.177	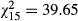 *P* < 0.001	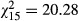 *P* = 0.161	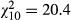 *P* = 0.026
(i) Chalkbrood strain I patriline effect	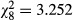 *P* = 0.978	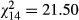 *P* = 0.089	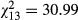 *P* = 0.003	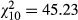 *P* < 0.001
(j) Number of patrilines	9	16	16	13
(k) Individuals genotyped	404	612	614	698
(l) HR (mean ± SE)	1.65 ± 0.12	1.93 ± 0.18	1.94 ± 0.09	2.28 ± 0.25

In addition to the total number patrilines identified through microsatellite genotyping, the total number of individuals genotyped, and the average (±SE) hazard ratio (HR) of each of the patrilines in each colony, as compared to survival of control individuals, based on the survival analyses.

**Figure 2:**
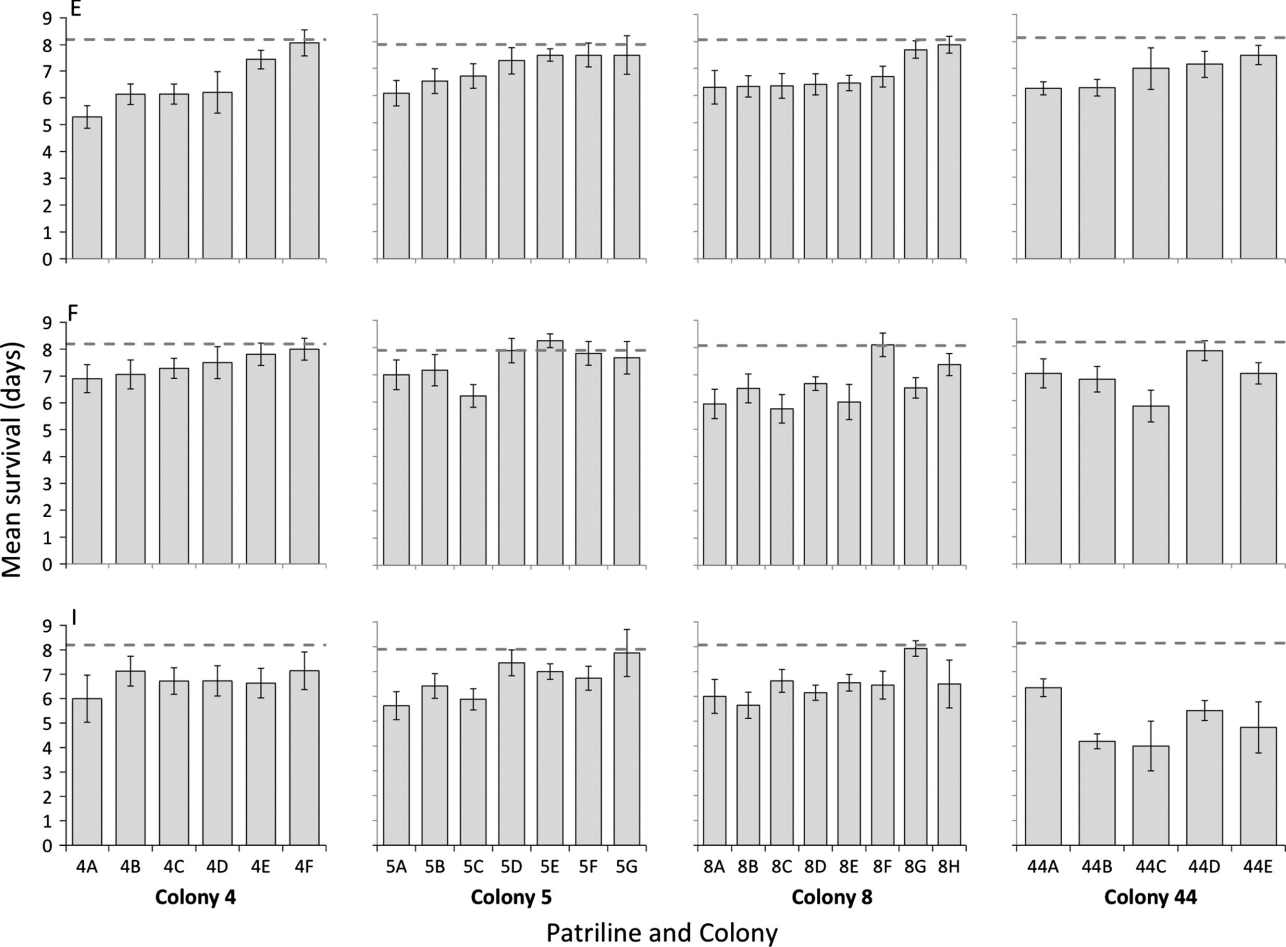
Mean ± SE survival time in days of each patriline within the four colonies (columns) split by chalkbrood, *Asc. apis* strains E, F, and I (top, middle, and bottom rows, respectively). Only patrilines containing more than three individuals per patriline per treatment are shown. The dashed line represents mean control survival level of that colony.

We then went on to look at the genotypic interactions between host and parasite by comparing survival after infection by the three different chalkbrood strains. Here, we found significant differences in survival after infection by the three different chalkbrood strains in three of four colonies, showing that colonies varied in their relative susceptibility to these strains (Fig. 2, row e). However, in only one of these colonies was there a significant interaction between host patriline and chalkbrood strain (Fig. 2; Table 1 row f), indicating relatively similar levels of resistance by host genotype to each of the three parasite genotypes. Only Colony 44 showed significant differences in survival between patrilines after exposure to all three strains, and it was this colony that also showed the interaction between patriline and chalkbrood strain in survival (Table 1 row f). The three other colonies we studied showed patrilineal differences in survival with only one strain: Colony 4 with strain E, Colony 5 with strain F, and Colony 8 with strain I, which also explains the lack of a significant interaction between patriline and strain in these three colonies.

Control survival was high in all colonies and the hazard ratios of exposure to the parasites corresponded to a medium to large effect size (Bedard et al. 2007). There were no significant differences between colonies in their average hazard ratios (χ^2^ = 5.82, df = 3, *P* = 0.121), showing that colony differences in patriline number, sample size, health, etc., did not significantly alter colony-level susceptibility in this experiment or the strength of G × G effect.

## Discussion

Here, we investigated the genetic basis to resistance to fungal brood parasites by the honey bee *A. mellifera*. We found significant variation in resistance patterns dependent on both host and parasite genotype, indicating a foundation for dynamic coevolutionary relationships between these species, and further support for the hypothesis that polyandry has evolved in part due to pressure from parasites. Our results corroborate previous studies on genetic resistance to brood diseases in the honey bee (Palmer and Oldroyd 2003; Tarpy 2003; Tarpy and Seeley 2006; Invernizzi et al. 2009). However, in this study we removed environmental effects as well as behavioral defenses in response to infection through our controlled laboratory infections. We did this in order to examine the specific dynamics between individual host and parasite genotypes and to assess the framework for coevolution between these species, particularly between the parasite genotypes of the obligate parasite chalkbrood. Our results are not attributable to intrinsic differences in survival between host genotypes as our control survival was always very high. Social insects combat disease using both behavioral and physiological mechanisms. Avoidance of infection through mechanisms such as hygienic behavior can be specific with regard to different parasite species but nonspecific with regard to within-parasite species variation (Schmid-Hempel and Ebert 2003), whereas immune-level defenses are likely to closer encompass true genetically based interactions, as the specificity of host–parasite interactions is often proposed to occur at the level of parasite recognition (Lambrechts et al. 2005). It is important therefore to investigate individual-level responses to infection when studying genotypic interactions in organisms that exhibit social immunity.

In all colonies, we found that the relative resistance of host genotypes varied depending on the genotype of the parasite they were exposed to. Host genotypes that were relatively resistant to one parasite genotype were sometimes relatively susceptible to other genotypes. In models of host–parasite coevolution, particularly those of the matching alleles type (Hamilton 1993), specific responses such as these are generated by the interaction between genes in the host and genes in the parasite. Our findings appear to be closest to matching allele-type models; when we assessed the effect of both parasite species we found no pattern of host genotypes that were consistently resistant or susceptible. This is similar to the dynamics seen for example in *Daphnia* interacting with its bacterial parasite *Pasteuria ramosa* (Carius et al. 2001), and the bumblebee *Bombus terrestris* with its parasite *Crithidia bombi* (Schmid-Hempel et al. 1999). When we considered only the three strains of the chalkbrood parasite, however, we found evidence of consistent resistance by patrilines in three colonies, indicated by the non-significant interaction term, which points more toward gene-for-gene dynamics for chalkbrood infections, similar to those seen in many plants interacting with their fungal pathogens (Thompson and Burdon 1992), but also the sort of patterns that are typical of, and give rise to the genetic diversity required for coevolutionary dynamics (Agrawal and Lively 2002; Salathé et al. 2008). The differences between colonies in survival after infection by different strains of the chalkbrood parasite also suggest genetic influences in resistance from the queens of these colonies in addition to the variable paternal resistance genes.

The genetic variation in resistance to the parasites also depended on the parasite species, indicated by the significant interaction between parasite species and patriline. We found higher levels of variation in resistance to the strains of chalkbrood parasites than we did the stonebrood parasite. All four colonies exhibited significant genetic variation in survival after infection by the chalkbrood parasite, whereas in only one colony did we find significant genetic variation in survival after infection by the stonebrood parasite. Importantly, each colony responded differently to the different chalkbrood strains, and only Colony 44 showed patrilineal differences in survival after infection with all three strains. An explanation for this might lie in the evolutionary history of these two host–parasite relationships as well as the life history of the two parasites. The chalkbrood parasite *Asc. apis* is an obligate parasite of honey bee larvae whereas stonebrood is caused by *Asp. flavus,* a facultative pathogen of honey bees that also affects other hosts (Vojvodic et al. 2011; Foley et al. 2012). Both are common parasites (Evison et al. 2012), but stonebrood is considered to be a relatively rare disease of honey bees despite *Aspergillus* infections being known to kill honey bees in all stages of development (Gilliam and Vandenberg 1997); it is virtually ubiquitous in the environment but leads a predominantly saprophytic lifestyle (De Vries 2008). The coevolution between the obligate chalkbrood parasite and its honey bee host should undergo negative frequency dependent selection. Assuming that some degree of genetic matching is required for infection, obligate parasites will be under strong natural selection to infect common host genotypes. If infection reduces host fitness, these common host genotypes should decrease in frequency over time and be replaced by previously rare host genotypes (Jayakar 1970; Tellier and Brown 2007). Coevolving parasites therefore select for rare host genotypes and preserve genetic variation in the population (Clarke 1976; Bell 1982; Hamilton 1982, 1993; Nee 1989; Zhang et al. 2013). This is highlighted by our results, which show that there is a higher amount of genetic variation in resistance to the coevolved parasite compared to the facultative parasite.

These findings provide evidence for coevolutionary interactions between resistance in the host and virulence in the parasite, and a basis for the requirement for genetic variation in the host. Our results follow the dynamics predicted by the parasite/pathogen hypothesis (Hamilton 1987; Sherman et al. 1988) and support the hypothesis that females multiply mate in order to generate increased genetic diversity within their offspring to reduce parasite transmission by increasing the chance of resistance genes within the colony. Resistance to economically important honey bee parasites, such as the *Varroa* mite (Behrens et al. 2011) and American foulbrood (Palmer and Oldroyd 2003), can also have a genetic basis, which highlights the value of understanding disease dynamics for future bee breeding programs. The genetic variation that results from polyandry may enhance the disease resistance of colonies, but its benefits are likely to be of greatest importance under the real situation of multiple parasite pressures that honey bees are faced with.
